# Opportunistic Osteoporosis Assessment and Fracture Risk Determination Using Cancellous Density Measurement in Hounsfield Units of Native Lumbar Computed Tomography Images—A Comparative Study with Conventional Bone Density Evaluation

**DOI:** 10.3390/jcm14041226

**Published:** 2025-02-13

**Authors:** Julian Ramin Andresen, Guido Schröder, Thomas Haider, Reimer Andresen

**Affiliations:** 1 Division of Trauma Surgery, Department of Orthopaedics and Trauma Surgery, Medical University of Vienna, Währinger Gürtel 18-20, 1090 Vienna, Austria; thomas.haider@meduniwien.ac.at; 2 Department of Traumatology, Hand and Reconstructive Surgery, Rostock University Medical Center, Schillingallee 35, 18057 Rostock, Germany; guido.schroeder1@gmx.net; 3 Institute for Diagnostic and Interventional Radiology/Neuroradiology, Westkuestenklinikum Heide, Academic Teaching Hospital of the Universities of Kiel, Luebeck und Hamburg, Esmarchstraße 50, 25746 Heide, Germany; randresen@wkk-hei.de

**Keywords:** axial skeleton, fracture risk determination, Hounsfield unit, insufficiency fracture, bone density determination, bone mineral density, osteoporosis, QCT, CTXA

## Abstract

**Background/Objectives:** Osteoporosis is a global problem that will increase as the population increases and ages, requiring prevention, early detection, and appropriate treatment. An increasing loss in bone mineral density (BMD) is the hallmark of osteoporosis, leading to an increased risk for insufficiency fractures. We aimed to investigate and analyze the applicability of native lumbar spine computed tomography (CT) scans for the evaluation of bone density compared with standard bone density measurements with quantitative computed tomography (QCT) and computed tomography X-ray absorptiometry of the hip (CTXA). **Methods:** Patients who were referred to our institution for diagnostic investigations and underwent CT imaging of the lumbar spine, as well as standard osteoporosis assessments including QCT and CTXA, were included in the study, resulting in a total of 240 patients (mean age: 65.9 years, range: 24–91). An ANOVA test was used to compare patient groups without a fracture, with one fracture, with more than one fracture, and with additional sacral fractures. An ROC analysis was performed to assess the predictive power of fracture risk estimation considering HU, QCT, and CTXA values. **Results:** At least one fracture was detected in 42.9% of these patients. For the lumbar spine, the median HU was 89.9 (range 67.9–126.9) and the median BMD was 73.7 (range 57.1–104.2) mg/cm^3^. With a correlation coefficient of 0.98 (*p* < 0.001), the HU values obtained from native lumbar CT scans can be calculated using the following formula: BMDspine = 0.84 + (0.81 × HU). With HU values < 80 and a BMD of the lumbar spine < 66 mg/cm^3^, a significantly increased number of osteoporotic vertebral fractures were found in the mid-thoracic, thoracolumbar, and sacral regions with an effect size of 0.89. In 32 patients (13.3%), additional sacral fractures were found; these patients showed the lowest density values with a median HU value of 31.8 (12.7–58.2). An ROC analysis of HU revealed a 93% sensitivity for the coincidence of a vertebral fracture. There was no significant difference compared with the AUC of QCT (*p* = 0.395) for concomitant vertebral body fractures. CTXA values also allowed for risk assessment but showed a significantly lower AUC. We found a negative correlation of BMD with age and a positive correlation of BMD with body mass index. **Conclusions:** Cancellous density measurements in HU values can be effectively converted into quantitative BMD values in mg/cm^3^, enabling a reliable assessment of osteoporosis severity and fracture risk prediction. Further quantitative density evaluation of the hip does not add value to fracture risk assessment for the axial skeleton. Based on this study’s findings, using HU values in native CT of the lumbar spine alone offers a viable, opportunistic approach towards fracture risk evaluation of the spine.

## 1. Introduction

An increasing loss in bone mineral density (BMD) with subsequent degradation of the microarchitecture leads to osteoporosis and insufficiency fractures of the axial skeleton [1]. Every year, there are >1.4 million cases of clinically conspicuous osteoporotic vertebral fractures (OVFs) worldwide [2]. In terms of epidemiology, OVFs are the most common osteoporotic fractures, occurring in 30–50% of the population over the age of 50 [3]. Patients who have suffered an OVF have an up to five times higher risk of suffering another vertebral fracture within the next year [4]. In the long term, these fractures minimize quality of life and increase mortality [5,6].

Risk factors for OVFs include prior fragility fractures, advanced age, female sex, vitamin D deficiency, rheumatoid arthritis, corticosteroid treatment, immobilization, low body mass index (BMI), excessive alcohol consumption, nicotine abuse, and other secondary causes of osteoporosis [4,5,6,7,8,9,10,11,12,13]. The distribution of fractures in the axial skeleton shows a peak frequency in the mid-thoracic and thoracolumbar region [14], whereby the curvatures of the spine in the sagittal plane are of biomechanical significance [15]. Even with manifest osteoporosis, insufficiency fractures in the cervical section are rarely found due to their distinct microarchitecture and relatively high density; a fracture threshold value does not appear to be undercut here [16]. On the other hand, with increasing severity of osteoporosis, more insufficiency fractures are found in the sacral section [17]. Dual-energy X-ray absorptiometry (DEXA) was considered the gold standard for determining bone density and identifying osteoporosis [18], whereby a T-score of lower than −2.5 is defined as osteoporosis [19]. Alternatively, quantitative computed tomography (QCT) can also be used to determine the BMD while measuring the cancellous bone in the lumbar spine; here, a BMD < 80 mg/cm^3^ is defined as osteoporosis [20,21]. With computed tomography X-ray absorptiometry of the hip (CTXA), DEXA-equivalent values can be reliably determined [22].

The extent to which it is possible to estimate the severity of osteoporosis and the fracture risk in the spine by determining density in Hounsfield units (HU) should be examined in comparison with the QCT and CTXA hip data.

## 2. Materials and Methods

### 2.1. Study Design

This study includes a retrospectively examined patient population.

### 2.2. Patient Population

There were a total of 240 patients with an ⌀-age of 65.9 (min.: 24; max.: 91) years and a body mass index (BMI) of 26.7 kg/m^2^ (min.: 17.6; max.: 60.6), including 40 men with an ⌀-age of 68.5 (min.: 42; max.: 84) years and a BMI of 24.9 kg/m^2^ (min.: 17.6; max.: 33.8) and 200 women with an ⌀-age of 65.3 (min.: 24; max.: 91) years and a BMI of 27.1 kg/m^2^ (min.: 17.7; max.: 60.6), with the question of presence of osteoporosis (Table 1). The patients were assigned from outpatient clinics for bariatric surgery, gynecology, geriatrics, neurosurgery, orthopedics, and traumatology.

### 2.3. Bone Density Analysis

**Figure 1:**
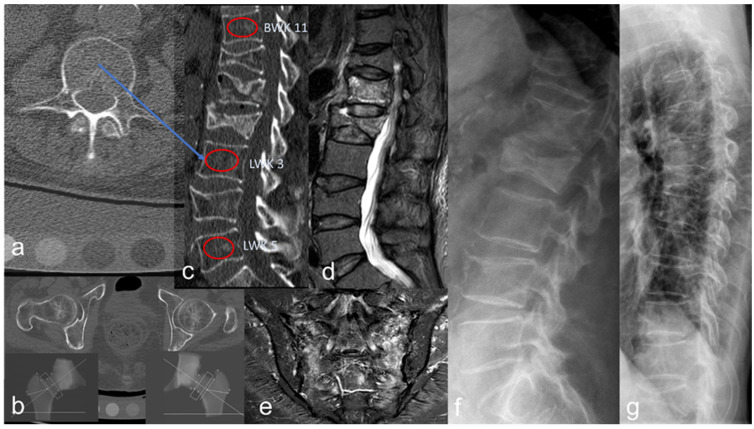
(**a**) Mid-vertebral axial CT segment with underlying reference body for lumbar osteodensitometry. (**b**) Axial and coronal CT segment from CTXA. (**c**) Sagittal mid-axial lumbar spine CT reconstruction with ellipsoid ROI drawn in the non-fractured vertebral bodies T 11, L 3, and L 5. OVFs are found at the level of T 12, L 1, L 2, and L 3. (**d**) The sagittal T2-weighted, fat-suppressed MRI segmented image shows clear signal enhancement, which is an expression of edema in the fresh sintering of the OVFs of L 1 and L 2. (**e**) Coronal MRI segmented image (STIR sequence) showing a bilateral fresh sacral fracture. (**f**,**g**) Lateral X-ray image of the lumbar spine and thoracic spine to detect the OVF.

The BMD was determined in mg/cm^3^ using QCT (GE-Revolution EVO/64 line CT and Mindways Software: 4.2.3, 3D Volumetric QCT Spine, Austin, TX, USA) in the vertebral bodies L 1, L 2, and L 3. After anonymization, an additional measurement of the cancellous bone density in HU was performed in the same vertebral bodies (720 vertebral bodies in total), in each case using an ellipsoid ROI manually positioned in the mid-vertebral cancellous space in the sagittally reformed CT segment, with a layer thickness of 2 mm and a window setting of C = 400/W = 1600 (Figure 1). In the presence of a pronounced degenerative vertebral body deformity, fracture, endospondylophyte, or hemangioma at the level of L 1, L 2, or L 3, an adjacent vertebral body was used for QCT and HU determination.

In addition, a BMD determination of the hip in mg/cm^2^ was performed using CTXA (Mindways software, CTXA, which uses the volumetric CT data set, Austin, TX, USA), and the corresponding T-score values were also included.

Mindways software is used worldwide and has the advantage where BMD can be evaluated independently of the CT type and specific company software.

### 2.4. Fracture Detection

In additional lateral radiographs of the thoracic spine (ThS) and lumbar spine (LuS), vertebral fractures were detected and graded according to Genant et al. [23]. This method considers the extent of vertebral height loss, with mild severity having an anterior, middle, or posterior height reduction of 20–25% (G1); moderate severity of 25–40% (G2) and severe severity of >40% (G3) compared with the adjacent vertebrae. In the case of clinically suspected insufficiency fractures in the sacrum, MRI segmented images performed at the same time were also evaluated.

### 2.5. Statistics

The collected data were analyzed using the statistical software package SPSS, version 23.0 (SPSS Inc., Armonk, NY, USA). For parametric tests, the quantitative characteristics were described as the mean (M), standard deviation (SD), and number (n) of available observations; they were presented using the interval mean ± standard deviation (M ± SD). For non-parametric tests, they were presented as the median with first and third quartiles (Q1–Q3). To describe a correlation between two variables, the Spearman correlation coefficient was calculated (r). A regression analysis was used to determine the QCT value (BMD-spine) and CTXA value (BMD-hip) using a generalized estimating equation. In addition, the correlation coefficients of HU values and QCT values were compared with the CTXA values. An ANOVA test was used to compare the patient groups without a fracture, with one fracture, with more than one fracture, and with additional sacral fractures. An ROC analysis was used to assess the predictive power with respect to fracture risk estimation, taking into account the HU, QCT, and CTXA values. For the determination of sensitivity and specificity, the confidence interval was set at 95%. All *p*-values are the result of two-sided statistical tests; in principle, *p* < 0.05 is considered significant. At the same time, the effect sizes were calculated according to Cohen, and values < 0.5 were assumed to be small, between 0.5 and 0.8 as a medium effect, and >0.8 as a large effect.

## 3. Results

**Figure 2:**
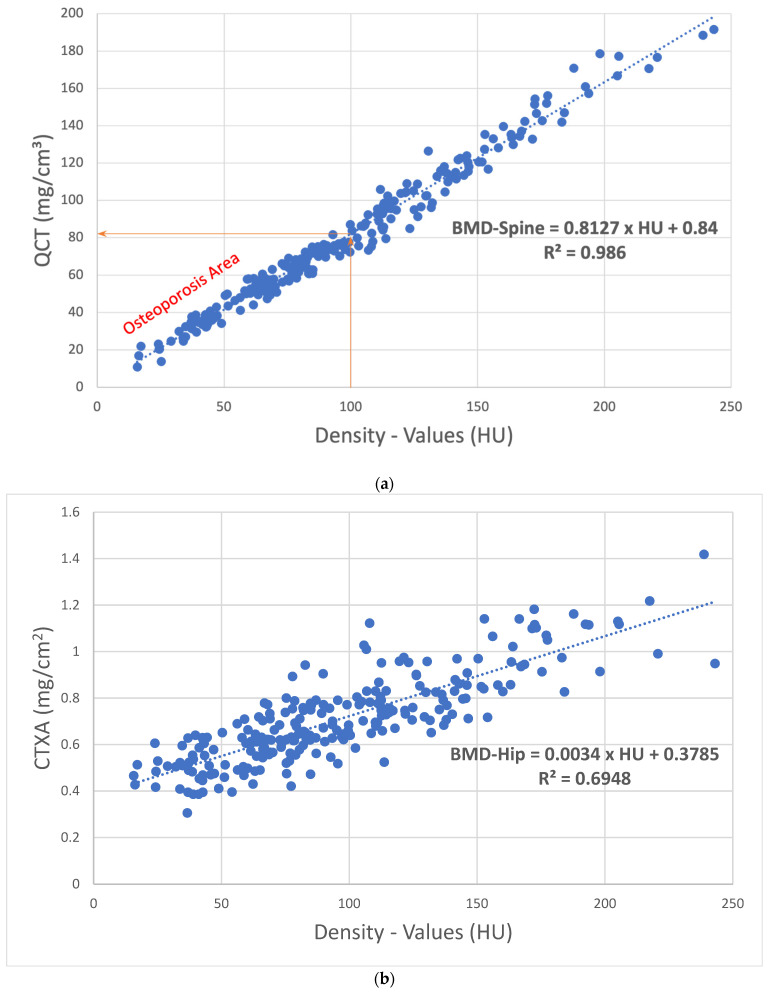
(**a**) Correlation of the cancellous density values in HU with the lumbar QCT values in mg/cm^3^. At 100 HU, the resulting BMD is 81.84 mg/cm^3^, which approximately corresponds to the defined osteoporosis limit [22], so that density values < 100 HU are in an osteoporotic range. (**b**) Correlation of the cancellous lumbar density values in HU with the CTXA values in mg/cm^2^.

The median HU was 89.9 (67.9–126.9) and median BMD was 73.7 (57.1–104.2) mg/cm^3^. With a correlation of R^2^ = 0.98 (*p* < 0.001), the HU values can be calculated using the following formula: BMD-spine = 0.84 + (0.81 × HU) can be converted to quantitative values in mg/cm^3^ (Figure 2a). Cancellous density values < 100 HU fit as a threshold for incipient osteoporosis (Figure 2a). With HU values < 79.8 and a BMD of the lumbar spine < 65.5 mg/cm^3^, significantly increased sintering fractures were found in the mid-thoracic, thoracolumbar, and sacral regions with an effect size of 0.89. At least one sintering fracture was found in 103/240 patients, whereby no fracture was detected in cranial of thoracic vertebral body (T) 5. In 32/103 patients, additional sacral insufficiency fractures were found; these patients showed the lowest density values with a median HU value of 31.8 (12.7–58.2) but did not differ significantly from the other patients with more than one fracture.

For CTXA, a correlation with the HU values of the lumbar spine of R^2^ = 0.69 (*p* = 0.238) was shown, whereby the HU values can be converted into quantitative values in mg/cm^2^ using the following formula: BMD-hip = 0.38 + (0.0034 × HU) can be converted into quantitative values in mg/cm^2^ (Figure 2b).

**Figure 3:**
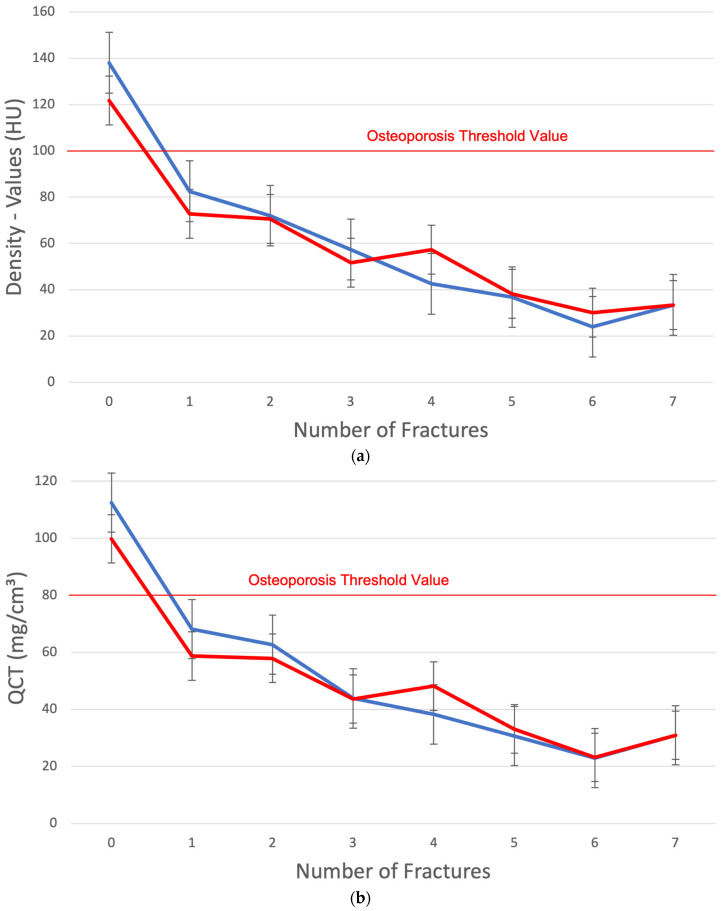

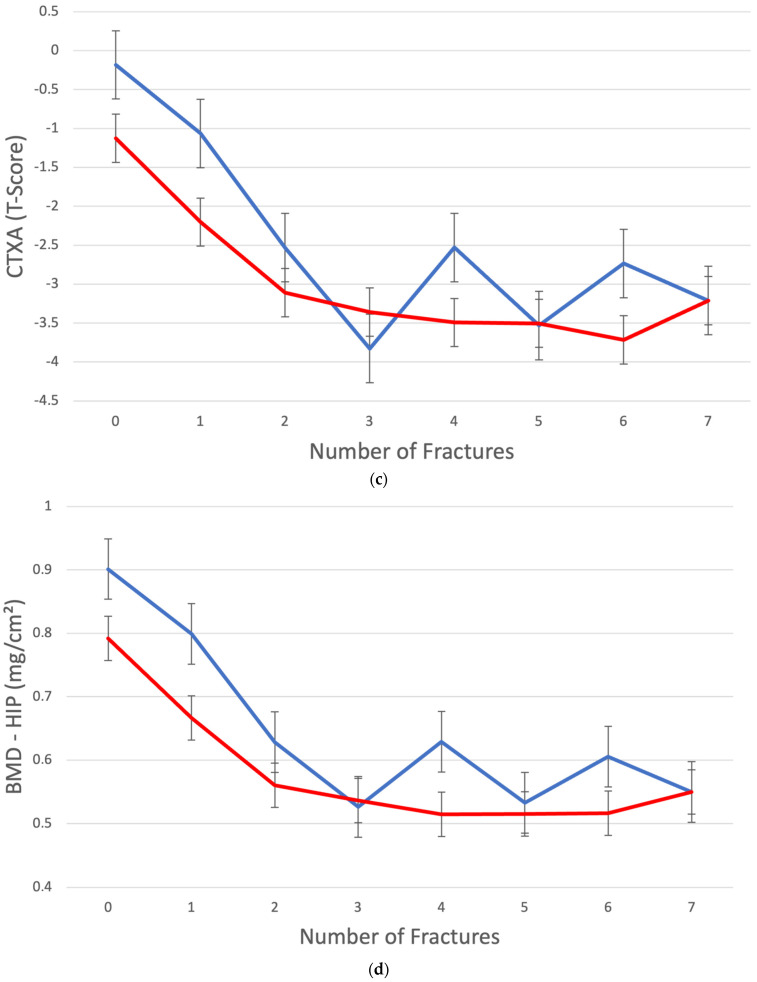
(**a**) Correlation between the cancellous lumbar density values in HU and the number of OVFs. The red line represents women, and the blue line represents men. There are no OVFs above the osteoporosis threshold of 100 HU. (**b**) The correlation between the lumbar QCT values in mg/cm^3^ and the number of OVFs. There are no OVFs above the osteoporosis threshold value of 80 mg/cm^3^. The red line represents women, and the blue line represents men. (**c**) Number of OVFs in relation to the CTXA (T-score). The red line represents women, and the blue line represents men. (**d**) Number of OVFs in relation to the CTXA (mg/cm^2^). The red line represents women, and the blue line represents men.

The mean values in HU show a clear decrease as a function of increasing fractures, with a fracture threshold value at <80 HU in men and <70 HU in women (Figure 3a). This corresponds with a fracture threshold < 65 mg/cm^3^ in men and <58 mg/cm^3^ in women in the QCT (Figure 3b) and with T-score values of the hip measurement <−1 in men and <−2.2 in women (Figure 3c). With more than one fracture, the HU is below 70 (Figure 3a), the QCT values are below 60 mg/cm^3^ (Figure 3b), the T-score values are less than −2.5 (Figure 3c), and the CTXA values are less than 0.7 mg/cm^2^ (Figure 3d).

**Figure 4:**
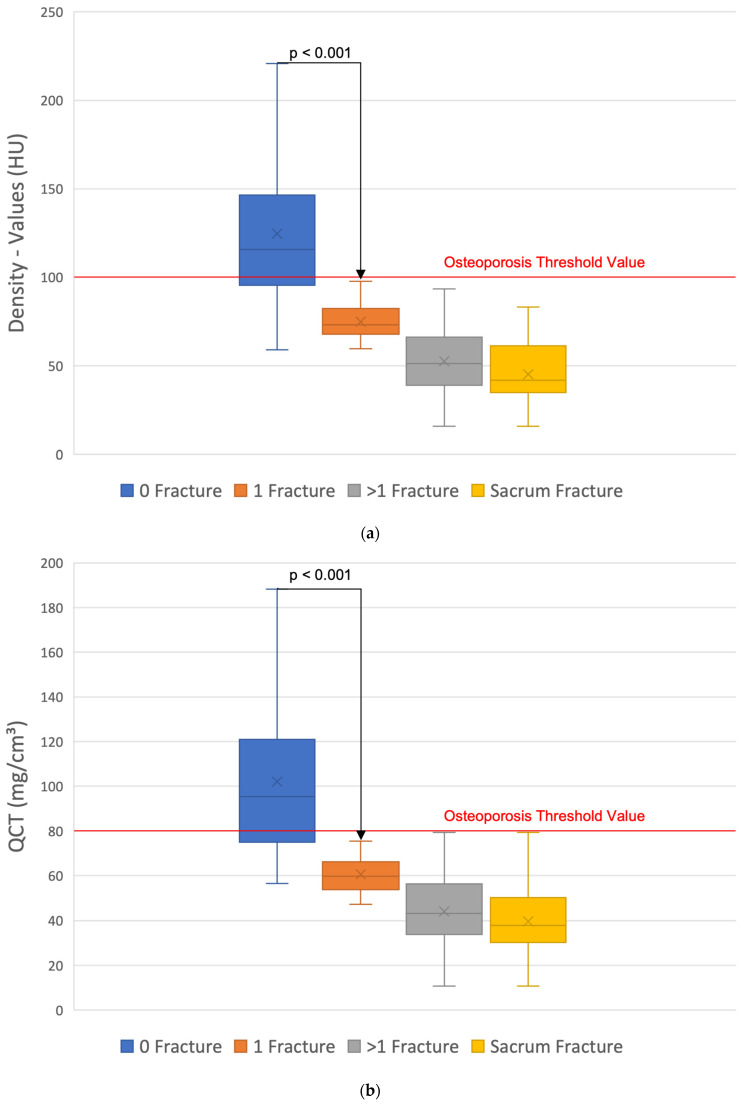

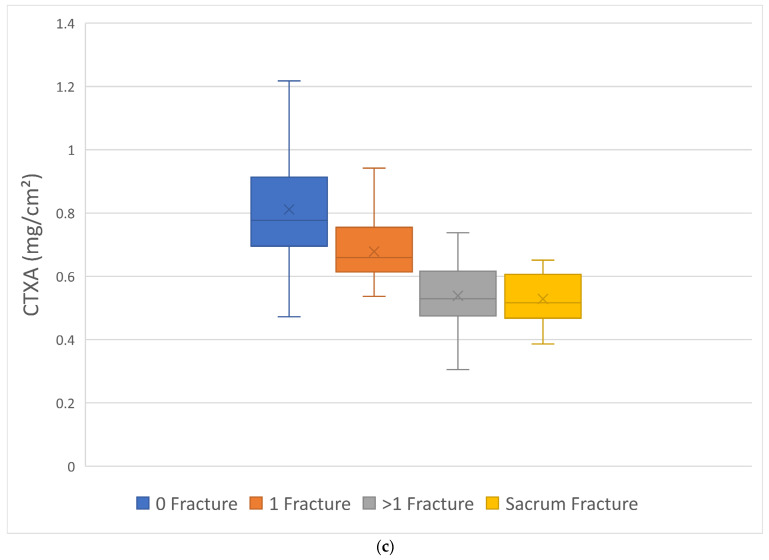
(**a**) The mean values of the density measurement in HU show a significant difference (*p* < 0.001) between patients without and with at least one OVF. Patients with sacral fractures have the lowest bone density. There are no fractures above the osteoporosis threshold value of 100 HU. (**b**) The mean values of the QCT values show a significant difference (*p* < 0.001) between patients without and with at least one OVF. Patients with sacral fractures have the lowest bone density. There are no fractures above the osteoporosis threshold value of 80 mg/cm^3^. (**c**) The mean values of CTXA in mg/cm^2^ show decreasing values with increasing number of OVFs.

Osteoporosis begins at a density of <100 HU; below <75 HU, the fracture threshold begins, and <60 HU fractures are significantly (*p* < 0.05) obligatory. Above 100 HU, there are no fractures in our patients (Figure 4). In the group without and with one fracture, there are individual overlaps in the density values, whereby the median values differ significantly with *p* < 0.05. In comparison with the group with more than one fracture, the difference becomes even clearer with *p* < 0.001. Patients with sacral fractures show the lowest cancellous density values, but there is no significant difference to the group with more than one fracture and without sacral fractures (Figure 4).

**Figure 5:**
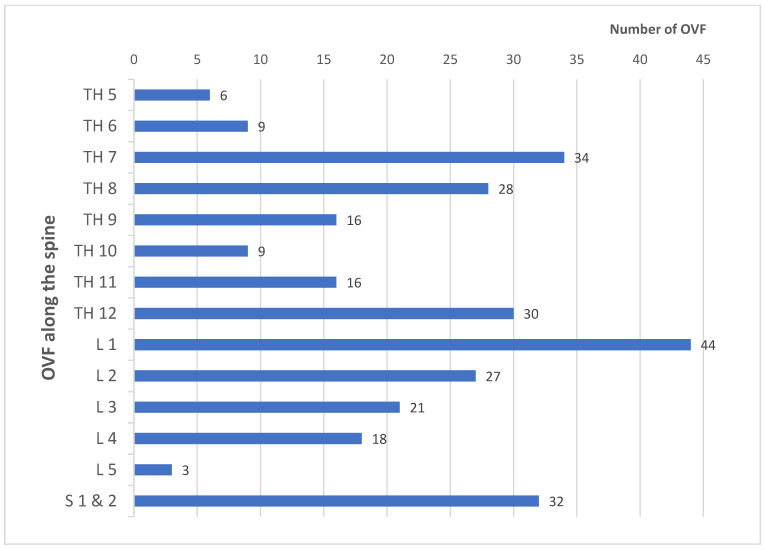
Distribution of OVFs along the spine. There are no fractures cranial of T 5. An accumulation of OVFs is found in the middle thoracic spine, thoracolumbar, and sacral regions.

The fracture distribution along the spine is shown in Figure 5.

**Figure 6:**
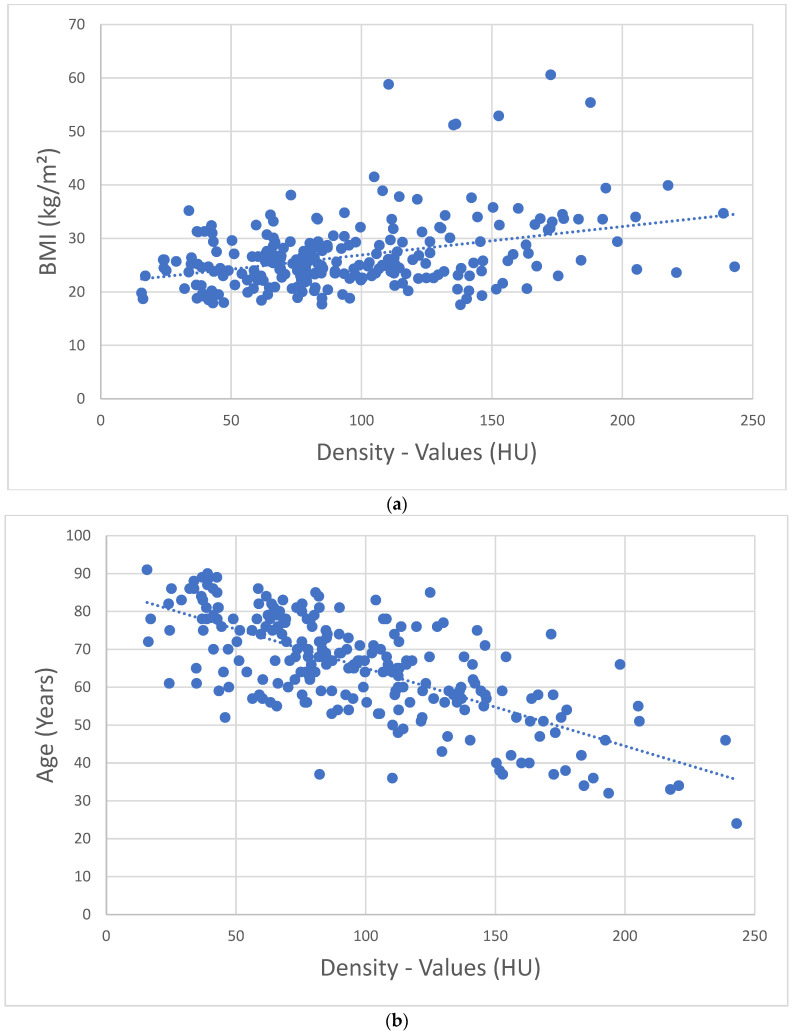
(**a**) Correlation of HU values and BMI. Patients with low BMI values have low density values (HU). (**b**) Correlation of HU values with patient age. Patients of advanced age have low density values (HU).

There is a direct correlation between density values in HU and the respective BMI; patients with a low BMI tend to have a low bone density (Figure 6a). An inverse correlation is found between HU density values and patient age, with older patients showing significantly (*p* < 0.001) the lowest density values (Figure 6b).

An overview of the patients and characterization of the total sample is shown in Table 1.

**Figure 7:**
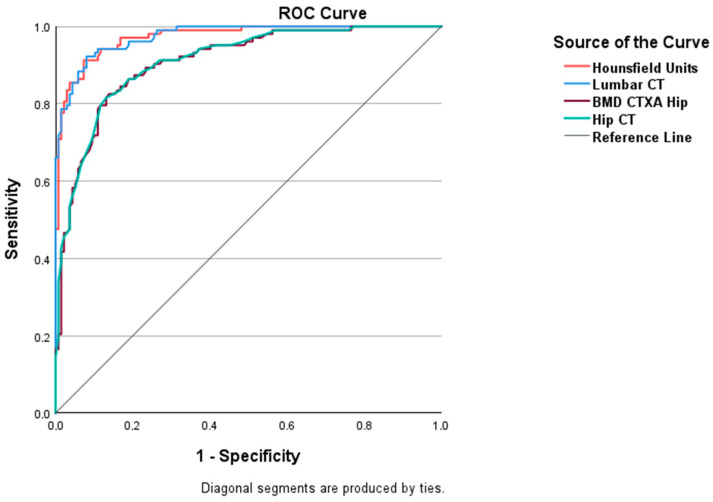
The ROCs curve analysis could not detect the AUC Hounsfield Units to be different from AUC lumbar CT (*p* = 0.395). Both the AUC of hip curves (CTXA in mg/cm^2^ and hip CT in T-Score) are comparable as well. A cut-off value of Hounsfield Units to optimally classify patients with and without fractures was determined by the maximal Youden index. Patients with ≤80 HU are assumed to have fractures (sensitivity = 0.89; specificity = 0.93). These diagnostic measures are comparable with those of lumbar QCT (sensitivity = 0.92; specificity = 0.92; cut-off ≤ 66 mg/cm^3^).

Using an ROC analysis, it can be shown that the significance for the risk of the occurrence of OVFs using HU values is very high at >93%, and there is no significant difference to QCT with *p* = 0.395. The CTXA values also allow for a risk assessment but show a significantly lower AUC (Figure 7).

## 4. Discussion

The cancellous density measurements in HU values can be converted into quantitative BMD values in mg/cm^3^ due to the high significant correlation, which allows for a good estimation of demineralization and severity of osteoporosis. The studies by Buenger et al. [24] also support this, whereby a high correlation was found between the HU from native CT sectional images and QCT values in 369 patients with a similar average age as our patients. In an in vitro study on 22 older body donors, Schröder et al. [25] found a comparable correlation. The different conversion formulas from the three studies are listed in Table 2; percentage differences in the conversions from HU to QCT values in mg/cm^3^ are shown for 100 HU as an example. A downward deviation of 9.9% in Schröder et al. [25], compared with their own collective, is likely due to an existing fat error [20,26], so that an overestimation of demineralization was found in the older body donors with manifest osteoporosis. If, in individual cases, diagnosis by HU determination is not possible with certainty, this could be corrected by an additional dual-energy quantitative computed tomography (DEQCT) [20,27]. Density determination in routine contrast-enhanced CT examinations is feasible. However, intravenous contrast agents can cause mean density increases of 8 to 30% [28,29,30], which must be considered when assessing osteopenia or osteoporosis. Buenger et al. [31] found a high correlation (r = 0.894, *p* < 0.001) between cancellous density values in HU measured in contrast-enhanced CT sections and previously or subsequently obtained QCT values in mg/cm^3^, which is considered in a practicable conversion formula (QCT values = 0.71 × HU + 13.82). No significant difference is to be expected in the cancellous density determination (HU) with experienced examiners, which was verified in an in vitro [25] and in vivo study [32]. The ROIs used should be standardized in shape and size.

Overall, low BMI (Figure 6a) [25], increasing age (Figure 6b) [25,33], and increasing fracture numbers show a significant decrease in bone density values (Figure 4) [34]. Regarding the discrimination of patients with and without OVF, Zou et al. [34] found results comparable with ours. The cancellous density < 70 HU is likely to be an OVF. According to BMD-spine = 0.84 + (0.81 × HU), 70 HU results in 57.5 mg/cm^3^, which is in good agreement with an earlier QCT evaluation, where 60 mg/cm^3^ is given as the fracture threshold [1]. Furthermore, 100 HU corresponds to 81.8 mg/cm^3^, which fits well with the established quantitative osteoporosis threshold of 80 mg/cm^3^ [20,21]. Lee et al. [35] found a cut-off value for existing osteoporosis of <110 HU at the level of L 1 in 94.5% of cases on sagittally reformed CT sections of routine contrast-enhanced CT examinations. Pickhardt et al. [36] found a threshold value for osteoporosis of < 110 HU with a specificity of 90% when measuring HU in the axial cross-sectional image. The cancellous axial HU values tend to be slightly higher than the sagittal values by approximately 2.6–6.2% [34], although an earlier study found no differences between measurements in the axial, sagittal, and coronal planes [37]. Kim et al. [38] found cut-off values of 110 HU in the coronal plane, 112 HU in the axial plane, and 112 HU in the sagittal plane. With comparable correlations with QCT and DEXA values as well as the estimation of fracture risk, there are no overall differences in the validation. However, it must be critically questioned whether a generalized bone loss can be inferred from the measurement of only one vertebral body [28,35]. Accordingly, Scheyerer et al. [39] considered the assessment of HU on at least three different lumbar vertebral bodies to be important. In our opinion, it is less important which lumbar vertebrae is measured, but rather that it is free of degenerative, post-traumatic, osteolytic, or postoperative changes. Cancellous density measurement across the entire axial skeleton is generally appropriate, though different threshold values must be considered for the cervical, thoracic, lumbar, and sacral regions [40,41]. In the craniocaudal direction, this shows a decrease in density values along the spine with or without osteoporosis [40,41,42,43]. The fracture distribution of our patients (Figure 5) shows a comparable distribution with other in vivo and in vitro studies [14,40]. The lowest cancellous density values as an expression of severe osteoporosis are found in the lumbar spine with concomitant sacral insufficiency fractures. On the other hand, these fractures are a clear indicator of osteoporosis [44].

Quantitative density determination of the hip (CTXA) does not provide any additional benefits for the risk assessment of OVFs and is significantly inferior to the cancellous density determination in HU and QCT in the lumbar spine in terms of sensitivity and specificity (Figure 7).

### 4.1. Further Clinical Significance of HU Density Determination

Choi et al. [45] found that DEXA measurements with degenerative spinal changes tended to give density values that were too high, although density determination using HU tends to reflect the actual situation. CT-HU values can be used here as a supplementary method to correct spinal osteoporosis not diagnosed by DEXA [46,47].

HU thresholds can be helpful for the risk assessment of mechanical complications after spinal deformity surgery in adults [48,49]. The determination of HU in the spine also has an influence on the assessment of complications after spinal surgery. Screw loosening can be more accurately predicted using HU values compared with other methods [50,51]. Overall, it was demonstrated that measuring cancellous bone density in HU with CT scans represents a promising alternative to DEXA measurement and, in terms of accuracy for osteoporosis diagnostics, appears to be partially superior [52].

In patients undergoing percutaneous balloon kyphoplasty treatment for OVFs, low HU values are a predictor of subsequent fractures [53]. The increase in bone density after osteoanabolic therapy can also be reliably monitored using HU measurements [54].

### 4.2. Strengths and Weaknesses

The strength of the study is that the wide range of patient ages, BMI values, and presence of patients with and without manifest osteoporosis suggest a wide range of HU values and thus reliable correlations. The calculated fracture risk applies only to additional vertebral fractures and does not include fractures in other parts of the skeleton. To ensure comparable values across different institutions, HU measurements should be conducted under standardized conditions (a possible CT tube voltage of 120 keV, CT slice thickness of 2 mm, window setting of C = 400/W = 1600 and ROI comparable in shape and size). An automatic contour-finding program, which could be implemented technically without great effort, would be helpful in terms of accuracy for recording the cancellous space. Factors such as patient age, osteoporosis severity, BMI, and sex likely influence the conversion formula and should be accounted for when comparing with other studies (Table 2) [24,25]. Ideally, the findings from our retrospective study should be validated through a prospective, randomized, multicenter follow-up study.

## 5. Conclusions

Based on the results of this study, an opportunistic evaluation using only HU values in the native CT of the lumbar spine to assess osteoporosis and determine the risk of OVFs appears to be valid. Based on the literature data, this is also approximately possible in contrast-enhanced CT examinations. An additional QCT/DEQCT or DEXA of the lumbar spine can clarify the definitive diagnosis in borderline cases but no longer appears necessary in most cases if a CT scan has been performed, avoiding further radiation exposure and additional costs [52,55].

## Figures and Tables

**Table 1:** Patient description by sex, age, BMI, and number of OVFs. OVFs were detected and graded according to Genant et al. [23]. This considers the extent of vertebral height loss, with mild severity having an anterior, middle, or posterior height reduction of 20–25% (G1); moderate severity of 25–40% (G2); and severe severity of >40% (G3) compared with the adjacent vertebrae.

Characterization of the Total Sample			
	Patients (n = 240)	Men (n = 40)	Women (n = 200)
Age (in years)	⌀ 65.9	⌀ 68.5	⌀ 65.3
BMI (kg/m^2^)	⌀ 26.7	⌀ 24.9	⌀ 27.1
Fractures	total: 293 G1: 128 G2: 111 G3: 22 Sacrum: 32	total: 36 G1: 19 G2: 13 G3: 1 Sacrum: 3	total: 257 G1: 109 G2: 98 G3: 21 Sacrum: 29

**Table 2:** Comparison of own results with two methodologically comparable studies. With high correlation, the HU values can be converted into quantitative values (mg/cm^3^) in all studies. At a threshold value of 100 HU, deviating quantitative values can be seen, which are caused by different patient collectives. The older the patient collective, the more pronounced a possible fat error becomes, which could be corrected by a DEQCT in a borderline case [20,26].

Conversion Values of Different Studies			
Study Group	Average Age (in Years)	Conversion Formula from HU in mg/cm^3^	Absolute Value and Percentage Deviation at 100 HU Compared with Own Results
Own results	65.9	QCT-Value =0.84 + (0.81 × HU)	81.8 mg/cm^3^
Buenger et al., 2021 [24]	66.98	QCT-Value =17.8 + (0.7 × HU)	87.8 mg/cm^3^ about 7.3% higher
Schröder et al., 2023 [25]	81.1	QCT-Value =13.7 + (0.6 × HU)	73.7 mg/cm^3^ about 9.9% lower

## Data Availability

The raw data supporting the conclusions of this article will be made available by the authors on reasonable request.

## Abbreviations

The following abbreviations are used in this manuscript:

AUC	Area under the curve
BMD	Bone mineral density
BMI	Body mass index
CT	Computed tomography
CTXA	Computed tomography X-ray absorptiometry
DEQCT	Dual-energy quantitative computed tomography
DEXA	Dual-energy X-ray absorptiometry
HU	Hounsfield Units
LuS	Lumbar spine
L	Lumbar vertebral body
mg/cm^2^	Milligram/Square centimeter
mg/cm^3^	Milligram/Cubic centimeter
OVF	Osteoporotic vertebral fractures
QCT	Quantitative computed tomography
Pat	Patient
ROCs	Receiver operating characteristics
ROI	Region of interest
ThS	Thoracic spine
T	Thoracic vertebral body
